# Green Synthesis of Gold and Silver Nanoparticles Using Leaf Extract of *Clerodendrum inerme*; Characterization, Antimicrobial, and Antioxidant Activities

**DOI:** 10.3390/biom10060835

**Published:** 2020-05-29

**Authors:** Shakeel Ahmad Khan, Sammia Shahid, Chun-Sing Lee

**Affiliations:** 1 Center of Super-Diamond and Advanced Films (COSDAF) and Department of Chemistry, City University of Hong Kong, 83 Tat Chee Avenue, Kowloon 999077, Hong Kong; 2 Department of Chemistry, School of Science, University of Management and Technology, Lahore 54770, Pakistan; sammia.shahid@umt.edu.pk

**Keywords:** green synthesis, *C. inerme*, gold, silver, antibacterial, antimycotic, antioxidant

## Abstract

Due to their versatile applications, gold (Au) and silver (Ag) nanoparticles (NPs) have been synthesized by many approaches, including green processes using plant extracts for reducing metal ions. In this work, we propose to use plant extract with active biomedical components for NPs synthesis, aiming to obtain NPs inheriting the biomedical functions of the plants. By using leaves extract of *Clerodendrum inerme* (*C. inerme*) as both a reducing agent and a capping agent, we have synthesized gold (CI-Au) and silver (CI-Ag) NPs covered with biomedically active functional groups from *C. inerme*. The synthesized NPs were evaluated for different biological activities such as antibacterial and antimycotic against different pathogenic microbes (*B. subtilis*, *S. aureus*, *Klebsiella*, and *E. coli*) and (*A. niger*, *T. harzianum*, and *A. flavus*), respectively, using agar well diffusion assays. The antimicrobial propensity of NPs further assessed by reactive oxygen species (ROS) glutathione (GSH) and FTIR analysis. Biofilm inhibition activity was also carried out using colorimetric assays. The antioxidant and cytotoxic potential of CI-Au and CI-Ag NPs was determined using DPPH free radical scavenging and MTT assay, respectively. The CI-Au and CI-Ag NPs were demonstrated to have much better antioxidant in terms of %DPPH scavenging (75.85% ± 0.67% and 78.87% ± 0.19%), respectively. They exhibited excellent antibacterial, antimycotic, biofilm inhibition and cytotoxic performance against pathogenic microbes and MCF-7 cells compared to commercial Au and Ag NPs functionalized with dodecanethiol and PVP, respectively. The biocompatibility test further corroborated that CI-Ag and CI-Au NPs are more biocompatible at the concentration level of 1–50 µM. Hence, this work opens a new environmentally-friendly path for synthesizing nanomaterials inherited with enhanced and/or additional biomedical functionalities inherited from their herbal sources.

## 1. Introduction

Development of multi-drug resistance (MDR) in bacterial strains, including *Enterococci, Staphylococci, Klebsiella, Acinetobacter, Pseudomonas, Enterobacter* species, etc., has become a severe challenge [1]. These bacteria are displaying resistance to world-leading antibiotics, including Cephalosporins, Carbapenems, Vancomycin, and Methicillin [2]. Furthermore, some fungi, for example, *Candida* species, are also showing resistance to various antifungal drugs, such as azole [3]. These pathogenic microbes are causing life-threatening diseases, such as aspergillosis, candidiasis, pneumocystis pneumonia, sepsis, osteomyelitis, meningitis, cholecystitis, severe bacteremia, diarrhea, tuberculosis [4,5]. Numerous antimicrobial drugs have been developed; however, because of the emergence of MDR in pathogens, clinical efficiency of existing drugs is vulnerable. Microbial spices are showing resistance to antimicrobial medicines by enzymatic deactivation and altering the drug target sites, decreasing antibiotics cell wall permeability, and displaying efflux mechanisms [6]. According to the World Health Organization (WHO), current deaths due to microbial diseases are ~0.7 million per year, if we could not develop efficient drugs to control or destroy these pathogenic microbes, the death caused by microbial diseases may rise to ~10 million by 2050 [7]. Therefore, this has become obligatory to find out alternate routes to tackle these MDR pathogenic microbes.

Nanotechnology has gained much attention for confronting these challenges. Nanomaterials of metal (Au, Ag, Se, etc.) and their oxides (CuO, ZnO, NiO, MnO, etc.) have been exploited as antibacterial agents, targeted drug delivery vehicles, antimycotic agents, antioxidant agents, anticancer agents, etc. [8,9,10,11,12,13,14]. Among them, Au and Ag NPs are of high significance due to their unique properties. They are extensively employed as anti-inflammatory, antibacterial, and antifungal agents in the coating of catheters, disinfecting medical devices, antimicrobial filters, dental hygiene, eye treatments, and wound dressings [8,9]. With nanometer sizes, these NPs can easily penetrate cell walls and cell membranes of pathogenic microbes in comparison to conventional antibacterial and antifungal drugs. This is a critical factor for their superior antimicrobial properties.

Numerous approaches have been used for preparing these metal NPs. These include physical (laser ablation, arc discharging, photolithography, ball milling, etc.), chemical (sol-gel, solvothermal, co-precipitation, pyrolysis, chemical redox reaction, etc.) and biological (plants, fungi, bacteria, virus, yeast, etc.) methods [15]. Physical and chemical methods often involve the use of toxic chemicals and solvents, which could have a harmful impact on the environment. In fact, the presence of residual hazardous chemical species on the surface of the synthesized NPs cannot be removed easily and could prohibit their biological and clinical applications. Moreover, their production often demands more energy and is not easily scalable [16]. Therefore, the use of biological methods for synthesizing NPs has gained much consideration as an alternative because it uses natural resources and is believed to be more biocompatible [17]. Synthesis of NPs using plants has gained tremendous attention over the last five years. It has eradicated complex steps, including maintenance of microbial cell culture, prolonged incubation time, several purification steps, etc., required for NPs synthesis using microorganisms such as fungi, bacteria, and yeast. Moreover, the usage of plants is considered more effective, easily scalable, and economical than other biological methods [16,17].

So far, uses of different plants for the synthesis metal nanoparticles are employed mostly for the lower manufacturing cost, easy scalability, and environmental friendliness. However, it should be noted that many plants have intrinsic biomedical applications that stem from their biologically active components, including polyphenols, alkaloids, saponins, terpenoids, flavonoids, etc. Here, we propose that if plants with intrinsic biomedical applications are used for preparing metal NPs, the obtained NPs might be capped with some of the biologically active components and thus inheriting their biomedical functions. To implement this concept, we have chosen leaves of *C. inerme*, which has been widely used for treating venereal infections, cough, fever, skin diseases, microbial infections, rheumatism, leprosy, etc., as the raw materials for preparing Au and Ag NPs [18,19,20]. By reducing Ag^+^ and Au^3+^ using *C. inerme* extract, Ag and Au NPs capped with various functional groups are obtained. It was found that these *C. inerme* –derived NPs show much better performance compared to commercial as well as other reported plant-derived Au and Ag NPs in terms of antimicrobial, antioxidant, ROS generation performance. To the best of our knowledge, it is the first demonstration that gold and silver NPs prepared with a *C. inerme* extract can inherit active biomedical components of the plant. 

## 2. Materials and Methods 

### 2.1. Chemicals

Analytical grade chemicals and reagents were purchased from Sigma-Aldrich or Merck. Leaves of the plant “*Clerodendrum inerme*” were collected from the hall area of City University Hong Kong on September 10, 2019. *C. inerme* leaves were identified and authenticated by taxonomist Professor Mansoor Hamid, Department of Botany, University of Agriculture Faisalabad, Pakistan.

### 2.2. Plant Extract Preparation

**Figure 1:**
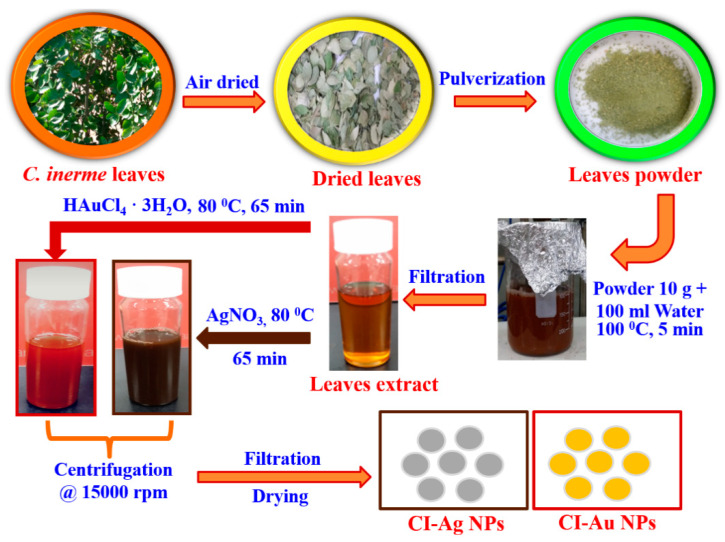
Schematic showing extract preparation and green synthesis of CI-Au and CI-Ag nanoparticles (NPs) using aqueous leaves extract of *Clerodendrum inerme*.

*C. inerme* leaves were first gently washed with deionized (DI) water to remove dust particles. Washed leaves were placed under shady areas for drying at room temperature (25–30 °C). Dried leaves were blended into powder with a commercial blender. After removing larger particles with a 200-mesh sieve (pore diameter of 0.074 mm), 10 g of the sifted leaves powder was added to 100 mL of DI water. The mixture was then heated to boil with continuous stirring for 5 min. The boiled mixture was further filtered with a sintered glass crucible to obtain a yellowish colored extract. The leaves extract of *C. inerme* was then stored in an airtight glass bottle placed in a refrigerator at 4 °C until further use (Figure 1).

### 2.3. Synthesis of CI-Au and CI-Ag NPs

The leaf extract was then used as a reducing and capping agent to convert gold and silver salts to their metallic forms (Figure 1). A total of 1 mM of, respectively, HAuCl_4_.3H_2_O and AgNO_3_ were added into 25 mL of leaves extract of *C. inerme*. The mixtures of gold and silver salts were both heated at 80 °C for 65 min with continuous stirring to obtain a ruby red and a dark brown dispersion of CI-Au and CI-Ag NPs, respectively (Figure 1). After centrifugation at 15,000 rpm for 15 min, the obtained CI-Au and CI-Ag NPs were washed with DI water three times and dried in an oven at 70 °C.

### 2.4. Characterizations

Standard transmission electron microscopy (TEM), X-ray diffraction (XRD), FTIR and UV-Vis spectroscopies, dynamic light scattering measurements were used for characterizing the compositions and structures of the synthesized CI-Au and CI-Ag NPs. Their performance in terms of antioxidant, antibacterial and antimycotic activities, and capability of reactive oxygen generation, as well as biocompatibility, were evaluated as described below. In addition to the CI-Au and CI-Ag NPs, for comparison, we also carried out the same measurements for the leaf extract, standard Au NPs functionalized with dodecanethiol (catalog no. 660434), and Ag NPs functionalized with PVP (catalog no. AGPB5-1M) purchased from Sigma-Aldrich and nanoComposix, respectively, as well as prototypical standard compounds including butylated hydroxytoluene (BHT, an antioxidant), Cephradine (an antibiotic drug), terbinafine hydrochloride (an antifungal medicine).

### 2.5. Antioxidant Activity

Antioxidant activity of the CI-Au NPs, the CI-Ag NPs, were determined by using a DPPH free radical scavenging assay [12]. In a typical experiment, 0.1 mM solution of DPPH was prepared in ethanol. Aqueous solution/dispersion of each sample with concentrations from 125 to 1000 µg/mL was prepared separately in order to evaluate concentration-dependent antioxidant potential. Each sample solution/dispersion was respectively mixed with a DPPH solution. The resultant reaction mixtures were stirred for 10 min at room temperature and set aside for 1 h. The antioxidant activity in terms of DPPH scavenging was then determined via the optical absorbance (As) at 517 nm measured with a UV-Visible spectrophotometer. Percentage of DPPH free radical scavenging was calculated by using the following equation:

% DPPH free radical scavenging = [(A_c_ − A_s_)/A_c_] × 100 (1)

where, A_s_ is the absorbance of the sample, and A_c_ is the absorbance of the control (only DPPH solution).

### 2.6. Antibacterial Propensity

The antibacterial propensity of all samples against two Gram-positive bacterial strains (*B. subtilis* ATCC 6051 and *S. aureus* ATCC 15564) and two Gram-negative bacterial strains (*Klebsiella* ATCC 13883 and *E. coli* ATCC BAA-196) were evaluated with the standard well diffusion method [21]. In a typical experiment, by means of a sterilized cotton-swab, a suspension of bacterial strains at a concentration of 5 × 10^5^ CFU/mL was swabbed onto Mueller-Hinton agar plates. Each sample of 50 µL with a concentration of 250 µg/mL was separately added into the wells of 6 mm in diameter. After hatching for 24 h at 37 °C, the sizes of bacteria inhibition zones on the plates were measured.

### 2.7. Minimum Inhibitory Concentrations 

The minimum inhibitory concentration (MICs) of the green synthesized CI-Au NPs, and CI-Ag NPs was determined in comparison with other samples following the protocol reported by [9] with slight modifications. In a typical procedure, the bacteriological strains at a concentration of 5 × 10^5^ CFU/mL were inoculated into 96-well plates. After that, 100 µL of Mueller Hinton broth containing different concentrations (40, 35, 30, 25, 12.5, 6.25, 3.125, 1.562, and 0.781 µg/mL) of each sample was serially diluted into a well of 96-well plates and incubated for 24 h at 37 °C. The 10 µL of 0.5% freshly prepared MTT (3-(4,5-Dime-thylthiazol-2-yl)-2,5-Diphenyltetrazolium Bromide) was added and incubated for 2 h in the dark. The 100 µL of DMSO (0.5%) was then added to solubilize the crystals of formazan and kept in the dark for 30 min. Finally, the optical density (OD) was measured at 595 nm wavelength to determine the percentage of bacterial cell death.

Percentage of bacterial inhibition = [(OD_control_ − OD_treatment_)*/*OD_control_] × 100 (2)

The MIC of each sample was determined based on the lowest concentration of the sample required to prohibit the 80% growth of the bacteriological strains.

### 2.8. Antimycotic Activity

The green synthesized CI-Au NPs, and CI-Ag NPs were assessed for their antimycotic activity against three pathogenic fungal strains (*Aspergillus niger*, *Aspergillus flavus*, and *Trichoderma harzianum*) using the standard agar well diffusion assay [22]. In a typical procedure, a freshly prepared autoclaved solution of potato dextrose (25 mL) was transferred to autoclaved petri dishes. Then 1 mL inoculum of already cultured fungal strains was transferred to each petri dish. Petri dishes were put aside for a while to allow solidification of the whole medium. Wells of 2 mm were bored from the solidified agar gel at four peripheral positions of each petri dish with a sterilized hollow iron tube. A total of 50 µL of each sample at 250 µg/mL was respectively added to the four wells on one petri dish. The Petri dishes were set aside for 1 h. Finally, the sizes of the fungal inhibition zone were measured after incubation at 25 °C for 24 h.

### 2.9. Biofilm Inhibition Activity

The green synthesized CI-Au NPs and CI-Ag NPs were assessed for their biofilm inhibition activity against pathogenic bacterial (*B. subtilis, S. aureus, Klebsiella,* and *E. coli)* and fungal strains *(Aspergillus niger, Aspergillus flavus,* and *Trichoderma harzianum*). In brief, biofilms of the microbial strains were developed using suitable media (TSB for bacteria and RPMI for fungi) in the 96-microtiter plate (10^7^ cells/well) at 37 °C for 24 h. The planktonic cells were then separated, and each well washed three times with PBS (phosphate buffer saline). After 50 µL of each sample at different concentration levels (0–120 µg/mL) was added separately into each well of 96-well plates. The 96-microtiter well plate was further incubated at 37 °C for 24 h. After drug treatment, the biofilms containing wells were gently rewashed with PBS. The staining agent, 90 µL of XTT, and 10 µL of phenazine methosulfate were added in each well and incubated in the dark at 37 °C for 4 h. The optical density (OD) was measured at 492 nm wavelength. The percentage of biofilm inhibition was calculated using the following equation

Percentage of biofilm inhibition = [(OD_control_ − OD_treatment_)*/*OD_control_] × 100 (3)

The minimum biofilm inhibitory concentration (MBIC) was determined as the lowest concentration of the drug molecule at which no biofilm formation of pathogenic microbes occur.

### 2.10. FT-IR Analysis of Bacterial and Fungal Strains

FTIR analysis of bacterial and fungal strains was carried out to identify molecular functionalities changes after their treatment with the green synthesized CI-Au NPs and CI-Ag NPs using FTIR spectrophotometer. At first, the greens synthesized NPs at the concentration of 250 µg/mL were employed to treat *E. coli* and *A. flavus* (5 × 10^5^ CFU/mL). Afterward, the microbial cells were obtained upon centrifugation (10,000 rpm, 15 min), and then their pellets formation (control and treated) was achieved upon treatment with KBr (1:100 ratios). Finally, they were analyzed using an FT-IR spectrophotometer.

### 2.11. Intracellular Reactive Oxygen Species (ROS) Analysis

Capability for producing intracellular ROS was investigated by employing 2′,7′-dichlorodihydrofluorescein diacetate (H_2_-DCFDA) as a probe, as described in the literature [23]. In a typical procedure, for microbial cells (E. coli and A. flavus) (10^5^ CFU/mL) incubation with a probe at 37 °C, 200 μM concentration of DCFH-DA was employed, followed by adding 50 µL of each sample at 250 µg/mL. After that, the incubation of microbial cells was further continued for 4 h at 37 °C. The results of intracellular ROS generation were recorded by measuring fluorescence emission at 523 nm and excitation at 503 nm using a Varian Eclipse spectrofluorometer. The results of NPs treated microbial cells were compared with 1 mM H_2_O_2_ treated (positive control) and untreated cells (negative control) to determine the ROS production capability.

### 2.12. Intracellular Glutathione (GSH) Investigation

The investigation of intracellular GSH production was performed following the procedure, as stated by Park et al., with minor amendments [24]. In a typical process, the microbial cells (10^5^ CFU/mL) were treated with 50 µL of each sample at 250 µg/mL, and subsequently, by using 5% TCA (trichloroacetic acid), they were lysed on ice for 15 min. After, 100 μL of cell lysate was treated with 900 μL of Tris−HCl (pH 8.3) and 100 μL of 1 mg/mL o-phthaldialdehyde solution. The resultant reaction mixtures were then subjected for incubation at 30 °C in the dark for 1:30 h. The fluorescence intensity of each sample with emission and excitation wavelengths of 420 and 350 nm, respectively, was recorded by employing the Varian Eclipse spectrofluorometer. The results of NPs treated microbial cells were compared with 1 mM H_2_O_2_ treated (Positive control) and untreated cells (negative control).

### 2.13. Cytotoxicity Activity 

The cytotoxicity activity of the green synthesized CI-Au, and CI-Ag NPs was determined against the MCF-7 cancerous cell lines compared to plant extract, Au, and Ag NPs following the MTT colorimetric protocol. The MCF-7 cancerous cells were placed in Dulbecco’s Modified Eagle’s Medium (DMEM) provided with streptomycin (100 µg/mL), penicillin (100 U/mL), and 10% FBS (fetal bovine serum) in a humidified atmosphere consisting of 5% CO_2_ and 95% air at 37 °C. The MCF-7 cancerous cells were cultured in 150 μL of DMEM in a 96-microtiter plate for 24 h at 37 °C in 5% CO_2_ to get cell-confluency up to 5 × 10^5^ cells/well. After 50 μL of NPs, plant extract, and standard drug at the concentration of 100 µg/mL were added separately in each well containing cultured cancerous cells, and the plate was then incubated for 24 h at 37 °C. Afterward, the cells were subjected to centrifugation for removing the supernatant, and then cells were washed with PBS solution. The 10 μL of MTT at 0.6 mg mL^−1^ concentration was added to each well, and the plate was further incubated again at 37 °C for 4 h. The DMSO at 100 μL volume was then transferred to each well for solubilizing the un-dissolved formazan crystals and placed the well plate on the shaker for 20 min. After, formazan’s absorption spectrum at 570 nm with reference at 655 nm was determined in each well employing the Varian Eclipse spectrophotometer and the cell viability (%) was calculated using the given formula:

Cell viability (%) = (OD_value of treated cells_)/(OD_value of negative control_) × 100 (4)

Treatment of cells with the standard drug (doxorubicin) was named as the positive control while cancerous cells without any treatment served as the negative control.

### 2.14. Biocompatibility

Hemolytic activity was carried out to determine the biocompatibility of the green synthesized CI-Au and CI-Ag NPs in comparison to the purchased metal NPs via the standard protocol, as reported by Khan et al. [18].

### 2.15. Statistical Analysis

All the experiments were repeated triplicates, and the results were presented as mean ± standard deviation. To determine the statistical difference, we have performed ANOVA analysis at a fixed significance level (0.05). Moreover, pairs Tukey’s test carried out to find out the significant pairs. 

## 3. Results

### 3.1. Compositions and Structures Analysis

**Figure 2:**
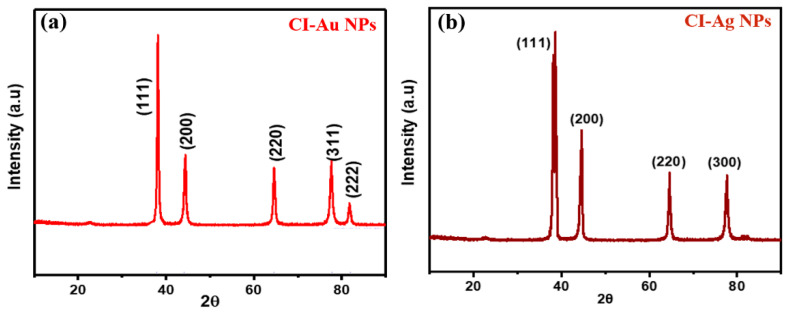
XRD patterns of green synthesized (**a**) CI-Au NPs and (**b**) CI-Ag NPs using leaves extract of *C. inerme*.

XRD patterns of the green synthesized CI-Au NPs, and CI-Ag NPs using *C. inerme* leaves extract are shown in Figure 2. The peak positions match well to those of metallic gold and silver, respectively, and show no observable impurity.

**Figure 3:**
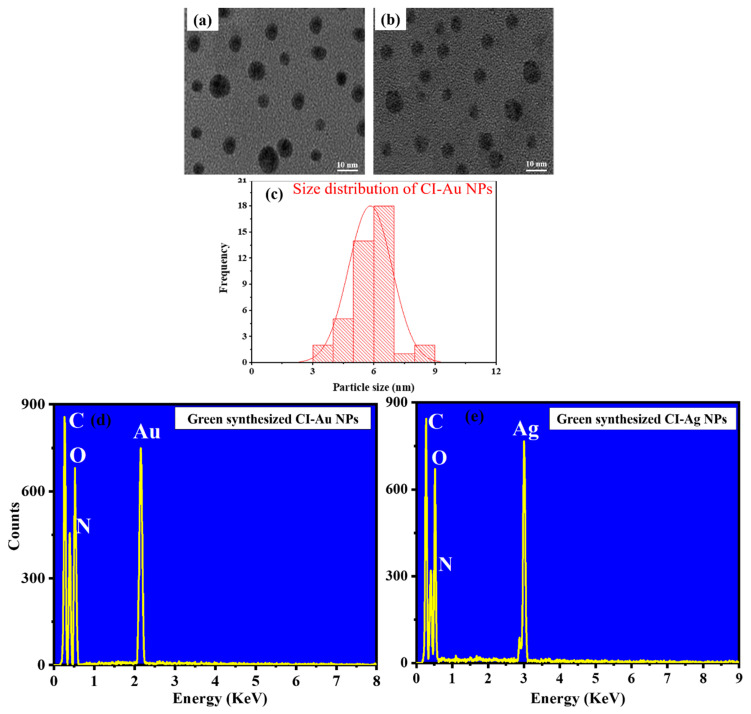
(**a**,**b**) TEM, (**c**) DLS, and (**d**,**e**) EDX spectra of green synthesized CI-Au and CI-Ag NPs, respectively, using leaves extract of *C. inerme*.

TEM images (Figure 3a,b) of the samples show that both samples are spherical in morphology, and they have average sizes 5.82 (CI-Au NPs) and 5.54 nm (CI-Ag NPs), respectively (Figure 3c). Further, the DLS particle size distribution of green synthesized CI-Au NPs, and CI-Ag NPs was also verified with the histogram generated by TEM (Figure S1). The composition of the NPs was measured using an energy dispersive X-ray spectrometer attached to the TEM. EDX spectra (Figure 3d,e) confirm that the two samples consist mainly of gold and silver, respectively. Peaks from C, O, and N are attributed to signal from the surface functional groups (e.g., polyphenols, flavonoids, proteins, etc.) on the nanoparticles as well as the holey carbon film, which holds the nanoparticle samples.

**Figure 4:**
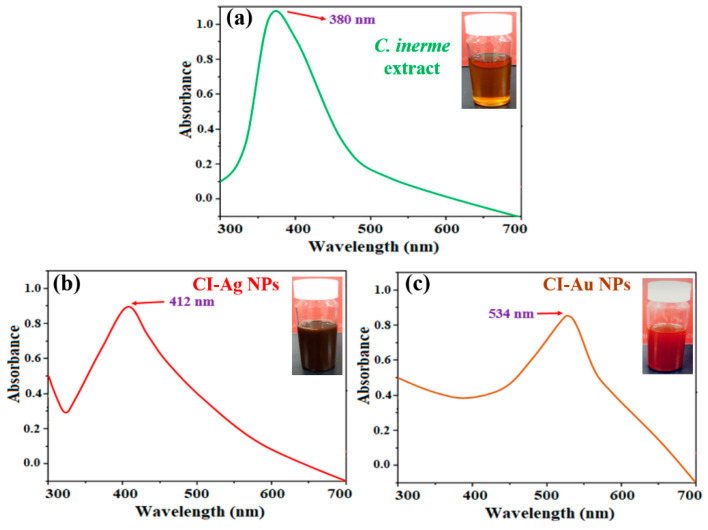
Absorption spectra of (**a**) extract of *C. inerme* leaves, (**b**) CI-Au NPs and (**c**) CI-Ag NPs.

Absorption spectra of the leaf extract and the nanoparticle are shown in Figure 4. Due to the surface plasmonic resonance phenomenon, the maximum absorption bands were observed at 534 nm for CI-Au NPs (Figure 4c) while for CI-Ag NPs at 412 nm (Figure 4b). Moreover, the leaf extract of *C. inerme* exhibited an absorption band at 380 nm (Figure 4a), which can be attributed to the absorption of polyphenols and flavonoids [9,25,26]. Alfuraydi et al. and Latha et al. reported the similar absorption spectrum for the green synthesized silver and gold NPs respectively [27,28].

**Figure 5:**
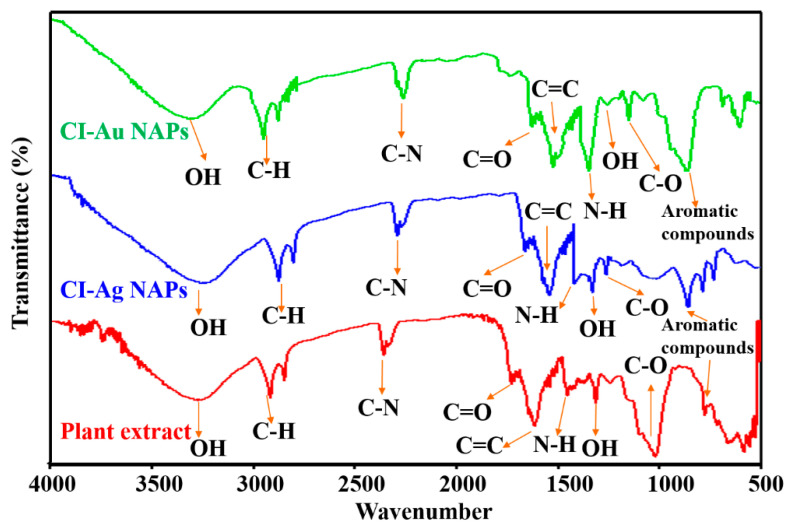
FT-IR spectra of green synthesized CI-Au NPs, CI-Ag NPs, and the *C. inerme* leaves extract.

FT-IR spectral study was carried out to characterize surface functional groups on the CI-Au NPs and the CI-Ag NPs. It can be seen from Figure 5 that types of nanoparticles show a FTIR signal corresponding to aromatic C=C (1520–1590 cm^−1^), C-H (2750–2860 cm^−1^), O-H (3310–3390 cm^−1^), N-H (1415–1490 cm^−1^), C–O–C (1025–1195 cm^−1^), C-N (2310–2350 cm^−1^), C=O (1690–1740 cm^−1^), O-H (1250–1310 cm^−1^), and aromatic compounds (675–815 cm^−1^). In fact, these match well to those in the FTIR spectrum of the *C. inerme* leaves extract. This suggests that many of the organic functional units in the leaf’s extracts are actually left on the surface of the CI-Au NPs and the CI-Ag NPs.

### 3.2. Antioxidant, Antibacterial, and Antimycotic Performance

**Figure 6:**
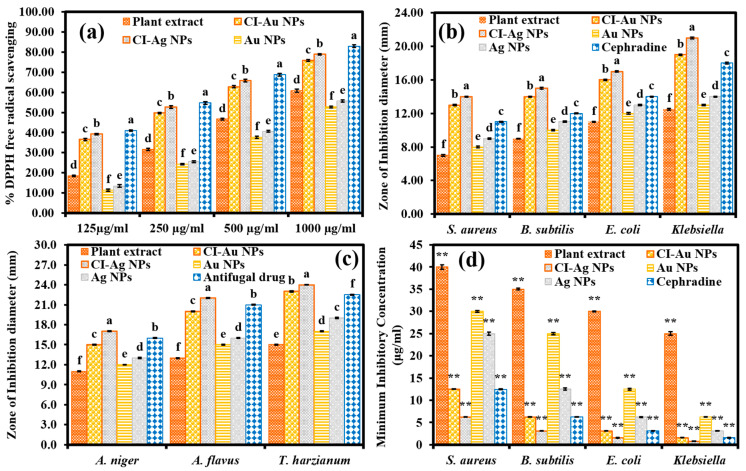
(**a**) Antioxidant, (**b**) antibacterial, (**c**) antimycotic activities, and (**d**) MICs results of green synthesized CI-Au NPs, and CI-Ag NPs in comparison to *C. inerme* leaves extract, Au NPs, Ag NPs, and standards (BHT, antibacterial and antifungal drugs). (Note; Tukey based heterogeneous lower-case letters represent significant statistical pairs). (** *p* < 0.01).

Antioxidant potentials of the CI-Au NPs and the CI-Ag NPs were determined by measuring their abilities to scavenge DPPH free radicals. Among all samples, the standard antioxidant BHT has the most substantial antioxidant capability (Figure 6a). The CI-Ag NPs and the CI-Au NPs show the second and third highest antioxidant capability, which are only slightly lower than that of BHT. It is interesting that the leaves extract shows higher antioxidant strength than the commercial Ag and Au NPs. These results suggest that the good antioxidant power of the CI-Au NPs and the CI-Ag NPs are likely to be associated with the leaves extract. We performed ANOVA test on DPPH scavenging results of six groups against different concentrations of 125, 250, 500, and 1000 µg/mL and the results revealed the statistical difference (F _(5,12)_ = 2043.11, *p* < 0.005), (F _(5,12)_ = 2081.81, *p* < 0.001), (F _(5,12)_ = 1755.24, *p* < 0.001), and (F _(5,12)_ = 1429.78, *p* < 0.001), respectively.

Antibacterial activity of all samples was compared via their zone of bacteria inhibition (ZOIs), as described in the experimental section. Figure 6b shows that for all the bacteria strains employed here, CI-Ag NPs and CI-Au NPs have the highest and the second uppermost antibacterial activities, respectively. It is impressive that their antibacterial performance is even better than the standard antibacterial drug Cephradine and the commercial Ag and Au NPs. Furthermore, we performed an ANOVA test on ZOIs results of six groups against each bacterial strain (*S. aureus*, *B. subtilis*, *E. coli*, and *Klebsiella*) and the results revealed the statistical difference (F _(5,12)_ = 3189.19, *p* < 0.001), (F _(5,12)_ = 3604.48, *p* < 0.001), (F _(5,12)_ = 2783.86, *p* < 0.001), and (F _(5,12)_ = 5454.22, *p* < 0.001), respectively. 

The antimycotic propensity of green synthesized CI-Au, and CI-Ag NPs was evaluated by using agar well diffusion assays against different pathogenic mycological strains. Figure 6c shows that the CI-Ag NPs have the best antimycotic performance for all the employed fungal strains. The performance of the CI-Au NPs and terbinafine hydrochloride are slightly below. Again, the antimycotic performance of the present CI-Ag and CI-Au NPs are much better than those of the commercial Ag and Au NPs. We performed an ANOVA test on ZOIs results of six groups against each fungal strain (*A. niger, A. flavus, and T. harzianum*) and the results displayed statistical significance (F _(5,12)_ = 4097.56, *p* < 0.002), (F _(5,12)_ = 10,326.18, *p* < 0.001), and (F _(5,12)_ = 8930.23, *p* < 0.001), respectively. 

The MICs generally employed to know about the minimum concentration of the drug molecule that required to prohibit the microbial growths. For this, four bacterial strains were evaluated using green synthesized CI-Au and CI-Ag NPs in comparison to the standard drug, commercial Ag, Au NPs, and plant extract. Figure 6d shows that CI-Ag NPs presented the highest antibacterial efficacy in terms of MICs compared to the standard drug, commercially purchased Ag NPs, Au NPs, and plant extract. On the other hand, the second-highest antibacterial performance in terms of MICs was exhibited by CI-Au NPs, which was comparable to standard drug. We carried out an ANOVA test on MICs results of six groups against each bacterial strain (*S. aureus*, *B. subtilis*, *E. coli*, and *Klebsiella*) and the results revealed the statistical difference (F _(5,12)_ = 3368.21, *p* < 0.001), (F _(5,12)_ = 3815.39, *p* < 0.005), (F _(5,12)_ = 3051.75, *p* < 0.001), and (F _(5,12)_ = 5649.33, *p* < 0.002), respectively. 

### 3.3. Biofilm Inhibition Activity

**Figure 7:**
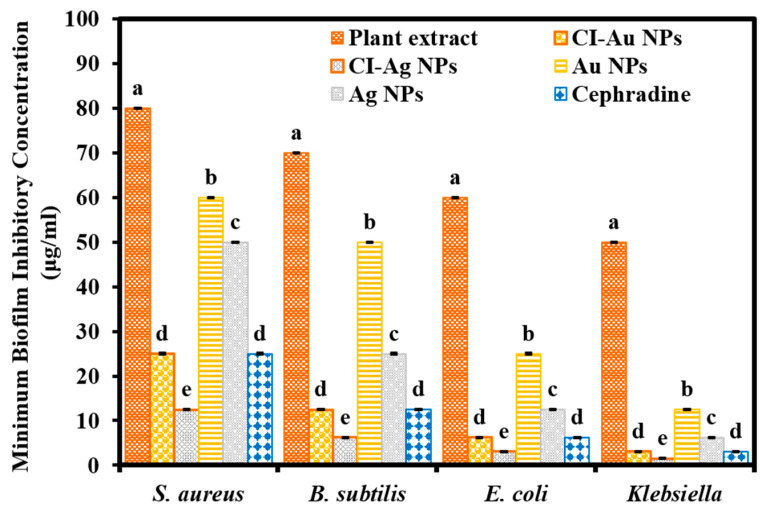
Biofilm inhibition activity of green synthesized CI-Au NPs, and CI-Ag NPs in terms of minimum biofilm inhibitory concentrations (MBICs) against different bacterial strains in comparison to *C. inerme* leaves extract, commercially purchased Au and Ag NPs, as well as Cephradine. (Note: Tukey based heterogenous lower-case letters represent significant statistical pairs).

**Figure 8:**
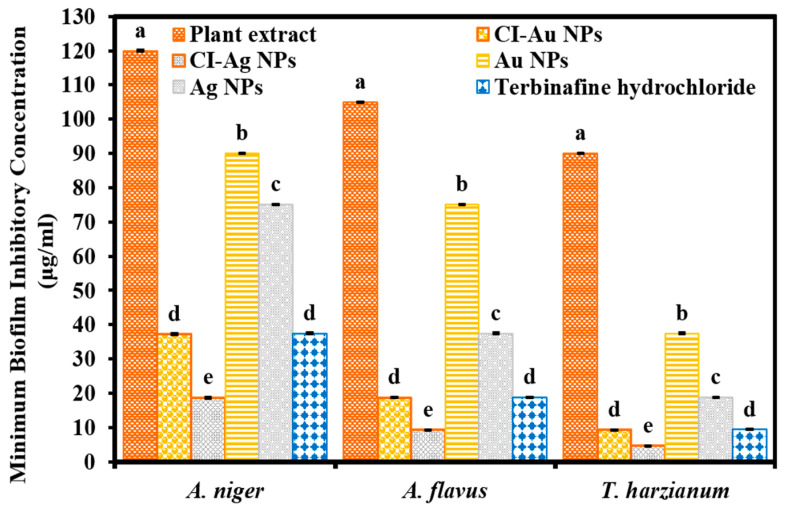
Biofilm inhibition activity of green synthesized CI-Au NPs, and CI-Ag NPs in terms of MBICs against different mycological strains in comparison to *C. inerme* leaves extract, commercially purchased Au and Ag NPs, as well as an antifungal drug. (Note: Tukey based heterogenous lower-case letters represent significant statistical pairs).

The biofilm inhibition ability of green synthesized CI-Au, and CI-Ag NPs was evaluated by using colorimetric assays against different pathogenic bacteriological and mycological strains, and their results in terms of MBIC are shown in Figure 7 and Figure 8, respectively. Results demonstrated that CI-Ag NPs and CI-Au NPs had demonstrated the highest and second uppermost biofilm inhibition activity against all the bacteria strains. It is impressive that the biofilm inhibition efficacy of CI-Ag NPs is even better than the standard antibacterial drug and the commercial Ag and Au NPs. While CI-Au NPs demonstrated comparable biofilm inhibition efficacy to the standard antibacterial drug (Figure 7). We performed an ANOVA test on the MBIC results of six groups against each bacterial strain (*S. aureus, B. subtilis, E. coli,* and *Klebsiella*) and the results revealed the statistical difference (F _(5,12)_ = 4415.21, *p* < 0.001), (F _(5,12)_ = 4705.24, *p* < 0.001), (F _(5,12)_ = 3817.71, *p* < 0.001), and (F _(5,12)_ = 5297.75.22, *p* < 0.001), respectively. 

Moreover, results were displayed that CI-Ag NPs have demonstrated excellent biofilm inhibition activity in terms of MBIC against all mycological strains than commercial Ag NPs, Au NPs, extract, and standard drug. While CI-Au NPs exhibited comparable biofilm inhibition activity in terms of MBIC to the standard antifungal drug but more significant than other samples (Ag NPs, Au NPs, and extract) (Figure 8). We performed an ANOVA test on MBIC results of six groups against each fungal strain (A. niger, A. flavus, and T. harzianum) and the results displayed statistical significance (F _(5,12)_ = 3161.91, *p* < 0.001), (F _(5,12)_ = 1454.04, *p* < 0.005), and (F (_5,12)_ = 2906.33, *p* < 0.001), respectively. A correlation has been observed in all results of antibacterial, antifungal, and biofilm inhibition activities.

### 3.4. The Capability of ROS Generation

**Figure 9:**
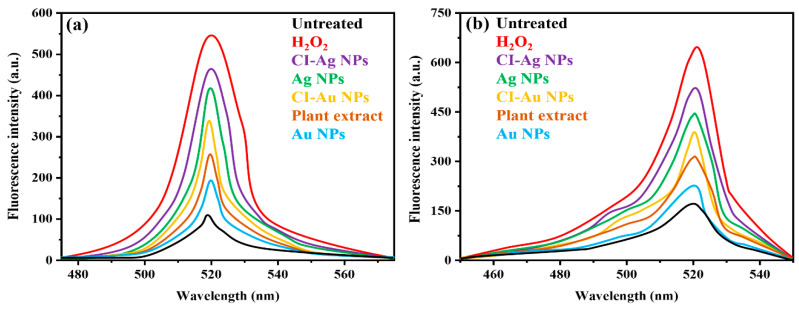
Fluorescent spectra measured with a confocal microscope from the H_2_-DCFDA probe inside (**a**) *E. coli* and (**b**) *A. flavus* cells after incubating with different samples.

It has been reported that oxidative stress plays a vital role in the annihilation of microbial strains [29]. Metal NPs interaction with microbial cells often generates ROS, including hydroperoxyl radicals (HO_2_^-^), hydrogen peroxide (H_2_O_2_), superoxide ions O_2_^−•^, and hydroxyl radicals OH^•^. They can induce oxidative stress inside the cell, leading to the destruction of various organelles and biomolecules. By using the H_2_-DCFDA assay, oxidative stresses in microbial cells (*E. coli* and *A. flavus*) after their treatment with the CI-Au and CI-Ag NPs were evaluated. H_2_-DCFDA will be oxidized in the presence of ROS and gives a green fluorescence peak at 503 nm upon photoexcitation. Figure 9 shows the fluorescent spectra of the H_2_-DCFDA probe inside *E. coli* (Figure 9a) and *A. flavus* (Figure 9b) cells after incubating with different samples. It can be seen that the ROS generation capabilities of the tested samples are in the order of H_2_O_2_ > CI-Ag NPs > Ag NPs > CI-Au NPs > plant extract > Au NPs > control (untreated) in both *E. coli* and *A. flavus*. In terms of ROS generation, silver NPs appear to have much better performance than gold NPs. Nevertheless, for both cases, those prepared with the *C. inerme* extract are better than the commercially purchased nanoparticles. This is hinting that the phytochemicals adsorbed on the surfaces of CI-Au and CI-Ag NPs are likely to contribute to the ROS generation.

### 3.5. Measurement of GSH Concentration

The capability of ROS generation was further corroborated by the quantification of produced intracellular GSH (a thiol-containing tripeptide) in microbial cells in response to oxidative stress results from NPs action. GSH is present in many of the microbial cells in its reduced form up to the concentration of 0.1–10 mM. GSH has the duty to provide protection to microbial cells against oxidative stress results from ROS and to maintain a cellular redox environment [30]. Therefore, it is very important for the survival of microbial cells to preserve naturally occurring antioxidant defense systems based on GSH. Nevertheless, upon GSH subjection to molecular oxygen, it oxidizes extemporaneously to disulfide (GSSG) (O_2_ + 2R−SH → RSSR + H_2_O_2_; ΔG_0_ = −96 kJ/mol) [31], thus upon enough GSH oxidation to GSSG leads to cell demise. Therefore, to understand the cellular oxidative stress in microbial cells upon treatment with the CI-Au NPs and the CI-Ag NPs, the intracellular GSH concentration was examined.

**Figure 10:**
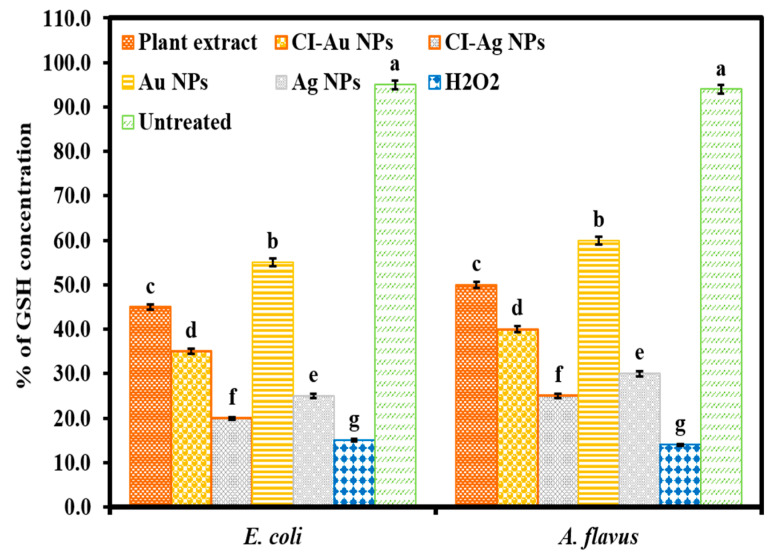
The level of GSH concentration in *E. coli* and *A. flavus* treated with *C. inerme* leaves extract, CI-Au NPs, CI-Ag NPs, Au NPs, and Ag NPs. (Note: Tukey based heterogeneous lower-case letters represent significant statistical pairs).

GSH concentrations measured from cells immediately taken out of the CO_2_ incubator are considered as 100%. GSH concentrations in cells incubated with different samples for 4 h at 37 °C were measured (Figure 10). The “untreated” samples referred to cells incubated without adding any of the NPs, H_2_O_2_ nor plant extract, and show GSH concentration of 95% and 94%, respectively, in untreated *E. coli* and *A. flavus*. This shows that under standard laboratory lighting of 4 h, a few percent of GSH were oxidized. The GSH concentration was reduced considerably to 45%, 35%, 20%, 55%, and 25% in E. coli and 50%, 40%, 25%, 60%, and 30% in *A. flavus* upon incubating, respectively, with *C. inerme* leaves extract, CI-Au NPs, CI-Ag NPs, Au NPs, and Ag NPs. As anticipated, the GSH concentration was attenuated remarkably to 15% and 14% in *E. coli* and *A. flavus*, respectively, in the presence of H_2_O_2_ (1 mM). We performed an ANOVA test on GSH concentration results of six groups against *E. coli* and *A. flavus*, and the results displayed statistical significance (F _(5,12)_ = 5944.36, *p* < 0.001), and (F _(5,12)_ = 4735.37, *p* < 0.001), respectively. These observations are consistent with the ROS generation results (Figure 9) showing that interaction of CI-Au NPs and CI-Ag NPs with microbial cells has developed the ROS mediated oxidative stress by depleting or destroying the antioxidant defense that leads to cell demise. Banerjee et al. has reported similar results upon E. coli treatment with iodinated chitosan−silver nanoparticle composite [32] and described that those NPs interact with microbial cells produced intracellular ROS mediated oxidative stress in the cell leading to cell membrane impairment and cell demise.

### 3.6. FTIR Analysis of Bacteria and Fungi

**Figure 11:**
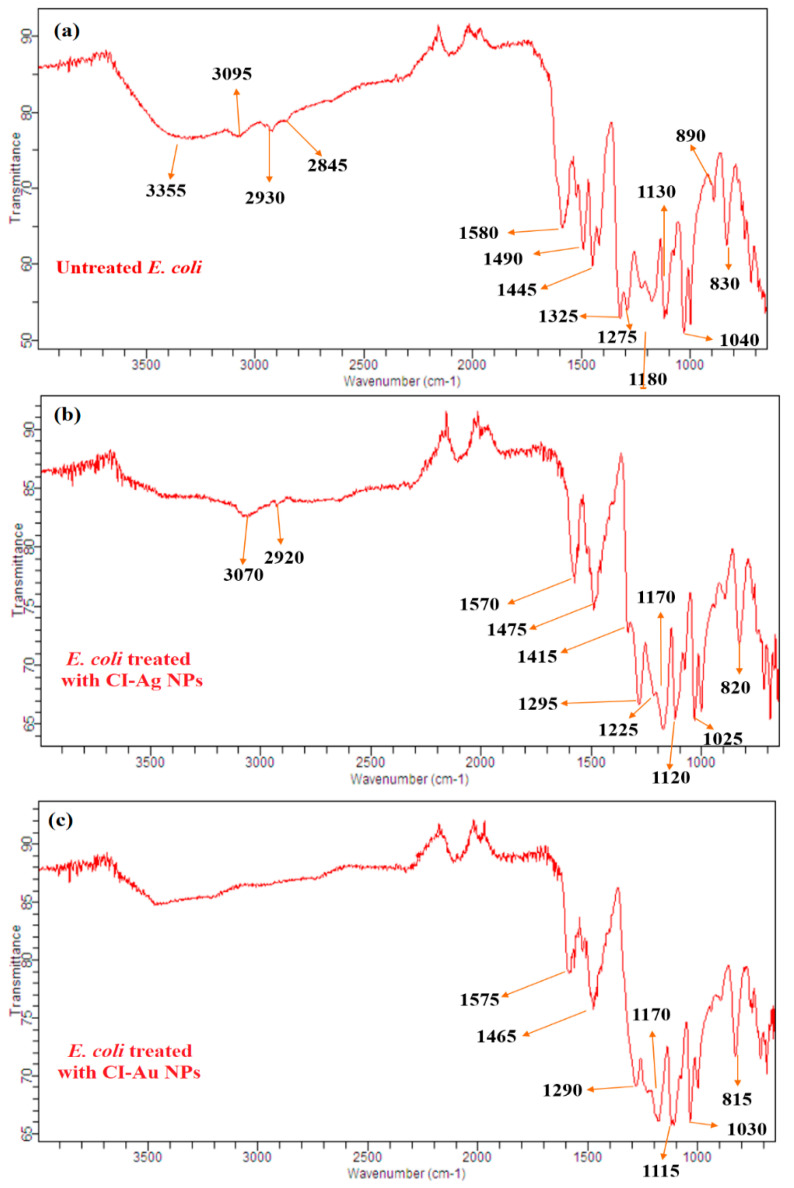
FTIR of (**a**) untreated and treated *E. coli* with (**b**) CI-Ag NPs and (**c**) CI-Au NPs synthesized by using *C. inerme* leaves extract.

**Figure 12:**
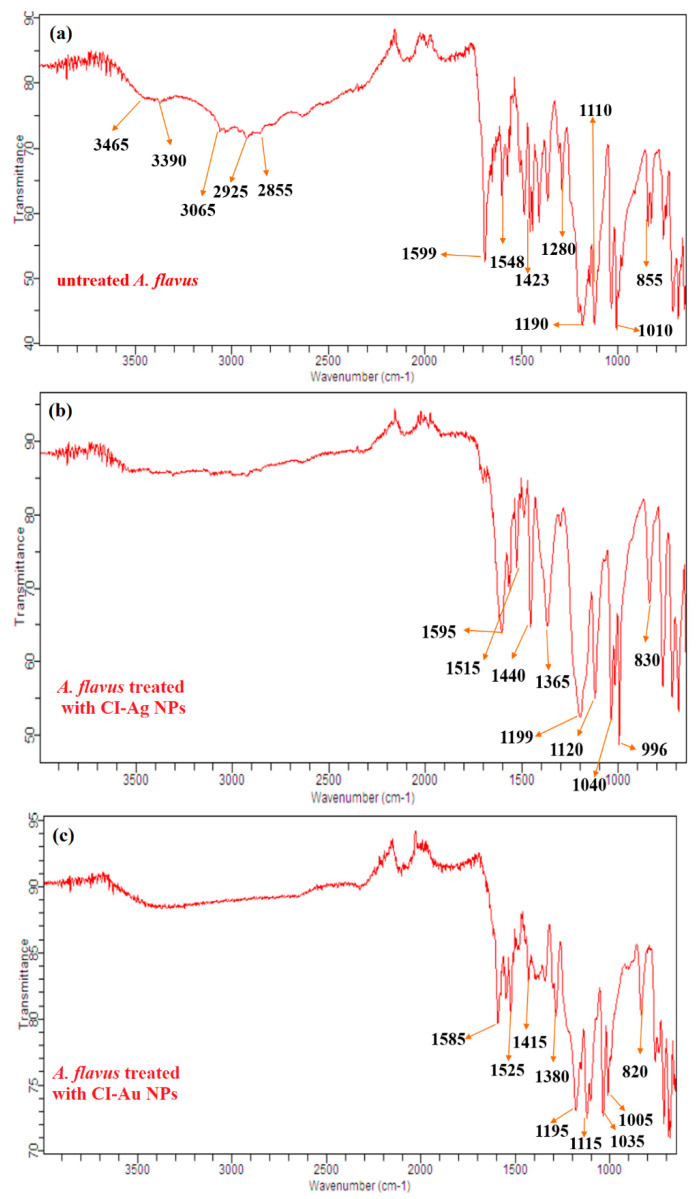
FTIR of (**a**) untreated and treated *A. flavus* with (**b**) CI-Ag NPs and (**c**) CI-Au NPs synthesized by using *C. inerme* leaves extract.

FTIR analysis of both untreated and treated microbial cells (*E. coli* and *A. flavus*) was performed to investigate further the binding of CI-Au NPs and CI-Ag NPs on their cell surfaces and subsequent changes in molecular functionalities of their cell membrane. Results demonstrated that the untreated bacterial cell (*E. coli*) showed characteristic FT-IR peaks (3335 cm^−1^ for O-H), (3095 cm^−1^ for -COOH), 2930 and 2845 cm^−1^ for C-H), (1580 cm^−1^ for amide I and amide II), (1490, 1445, and 1325 for -CH_2_), (1275 cm^−1^ for PO_2_^-^) and (1180, 1130, 1040, 890, and 830 cm^−1^ for C-O-C) from different proteins, fatty acids, and polysaccharide molecules present on the cell surface (Figure 11) [33]. The untreated mycological cell (*A. flavus*) showed FT-IR peaks from several functional groups, such as -NH_2_ (3465 cm^−1^), -OH (3390 cm^−1^), -COOH (3065 cm^−1^), aliphatic -CH (2925 and 2855 cm^−1^), amide I and amide II (1599 and 1548 cm^−1^), aromatic C=C (1423 cm^−1^), PO_2_^-^ (1280 cm^−1^), C-O-C (1190, 1110 cm^−1^), glucose ring band (1010 cm^−1^), and C-Cl (855 cm^−1^) (Figure 12) [34].

In contrast to untreated *E. coli*, the treated cells demonstrated the noticeable changes in whole FTIR spectral regions (Figure 11). The FTIR peaks shifting and reduction in band intensity were observed in treated microbial cells. The O-H peak at 3335 cm^−1^ in untreated *E. coli* disappeared in CI-Au NPs, and CI-Ag NPs treated bacterial cells. Moreover, the intensity of methylene and -COOH stretching vibrations were reduced in CI-Ag NPs treated and shifted to lower wavenumber, while their peaks were totally disappeared in CI-Au NP-treated *E. coli* cells. These observations demonstrate that fatty acids may undergo a number of significant reductions, which leads the *E. coli* cell membrane to transform from a well-ordered to disordered state [35,36]. The amide I and amide II peaks were shifted to downfield and appear at 1575 and 1570 cm^−1^ in *E. coli* cells treated with CI-Au NPs and CI-Ag NPs, which indicate changes in protein structure. This might be because of the cell membrane lysis of *E. coli*. The mild downfield shifting of -CH_2_ and C-O-C peaks were also observed in the treated *E. coli* cells. PO_2_^-^ peaks bands appeared strongly shifted in CI-Ag NPs treated cells but completely disappeared in CI-Au NPs treated *E. coli* cells. Both of these results evidently indicated that phospholipid molecules of the *E. coli* cell membrane were denatured upon processing with the CI-Au and CI-Ag NPs.

Similarly, CI-Au NPs and CI-Ag NPs treated *A. flavus* also showed noticeable changes in its FTIR spectrum (Figure 12). The -NH_2_, -OH, -COOH, aliphatic -CH, and PO_2_^-^ functional groups peaks appear in untreated *A. flavus* cells were utterly removed upon treatments with CI-Au NPs or CI-Ag NPs. In contrast to untreated *A. flavus* cells, FTIR peaks of amide I, amide II, Aromatic C=C, C-O-C, glucose ring band, and C-Cl are shifted higher and downfield following reduction in their intensity in CI-Au NPs, and CI-Ag NPs treated cells. In addition, some new peaks at 1365 and 1380 cm^−1^ are also observed in the FTIR spectrum of CI-Ag NPs, and CI-Au NPs treated *A. flavus* cells, respectively.

These FTIR results suggest that upon treatment with the CI-Au NPs and CI-Ag NPs, cell membranes microbial cells are destructed with the following changes: (1) well-ordered to disordered state transformation of cell membrane’s fatty acids as revealed by peaks intensity reduction and disappearance of OH, methylene, and COOH; (2) changes in the structure of membrane proteins shown by the downfield shifting of amide I and amide II peaks; (3) downfield shifting and disappearance of PO_2_^-^ peaks displaying possibly denaturation of phospholipid molecules; (4) obliteration of glycoside linkages (C-O-C) of membrane’s polysaccharide molecules [36,37,38,39,40,41].

### 3.7. Cytotoxicity Study

**Figure 13:**
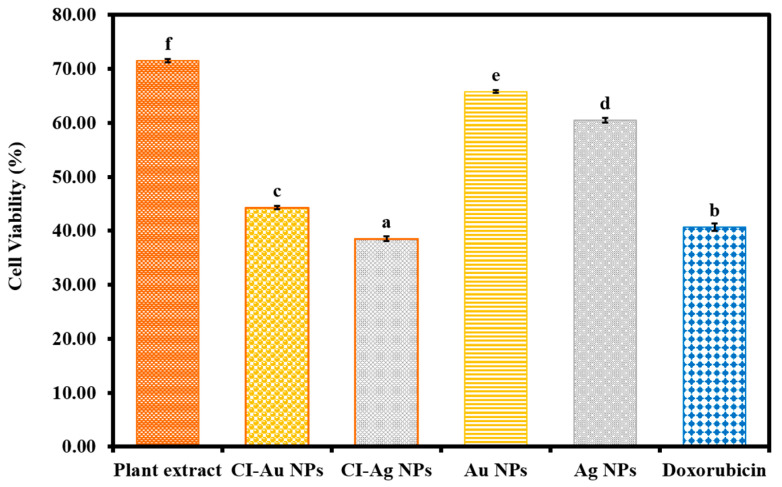
Cytotoxicity activity of green synthesized CI-Au NPs and CI-Ag NPs against the MCF-7 cancerous cells compared to *C. inerme* leaves extract, Au NPs, Ag NPs, and standard drug. (Note: Tukey based heterogeneous lower-case letters represent significant statistical pairs).

The green synthesized CI-Au and Cl-Ag NPs were evaluated for their cytotoxic propensity against the MCF-7 cancerous cell line in vitro compared to the *C. inerme* leaf extract, Au, Ag NPs, and standard drug. Figure 13 shows the cytotoxicity results. The results were demonstrated that green synthesized CI-Ag NPs displayed the superior cytotoxic effect on MCF-7 cancerous cells by lowering their cell viability percentage compared to other samples (Au, Ag NPs, and standard drug). While, the green synthesized CI-Au NPs have exhibited slightly lower cytotoxic propensity against cancerous cells compared to the standard drug but was higher than plant extract, Au, and Ag NPs. Interestingly, leaves extract of *C. inerme* also displayed cytotoxic potential against MCF-7 breast cancerous cells. Moreover, the statistical significance in the results of cytotoxicity activity of NPs, plant extract, and the standard drug was also validated with the ANOVA (F value = 3255.924, *p* < 0.001) and Tukey test (Figure 13). These good cytotoxicity activity results of green synthesized CI-Au, and Cl-Ag NPs might be attributed to their physical properties (small size, morphology, and surface area), and their functionalization with the biologically active phytomolecules of the leaves extract of *C. inerme*. Many reports disclosed that leaves extract of *C. inerme* have different phytomolecules that are biological active [18,19,20].

### 3.8. Biocompatibility Analysis

Biocompatibility of CI-Au and Cl-Ag NPs are assessed via their effects on red blood cells (RBCs). The hemolysis induced by the sample is presented in Figure S2. The ASTM international method was followed to determine whether the samples were hemolytic (0–2% non-hemolytic, 2–5% partially hemolytic, and ≥5% hemolytic).

The results show that CI-Au NPs and CI-Ag NPs were non-hemolytic at their lower concentrations (1 µM). However, they are partially hemolytic at higher concentrations (50 µM). On the other hand, the commercial Au and Ag NPs are both partially hemolytic even at a low concentration of 1 µM and fully hemolytic at a higher concentration (50 µM). The *C. inerme* leaves extract was demonstrated to be non-hemolytic at all tested concentrations. These results suggest that adsorbed phytochemicals on the surface of CI-Au NPs and CI-Ag NPs might lower the toxicity of the core metal nanoparticles. The statistical significance of the hemolytic activity results was further corroborated by the ANOVA (*p* < 0.001, F-value = 117,502.57) and Tukey test (heterogeneous lower-case letters) (Figure S2). Similar results were reported by Parthiban et al. [9]. 

## 4. Discussion

**Figure 14:**
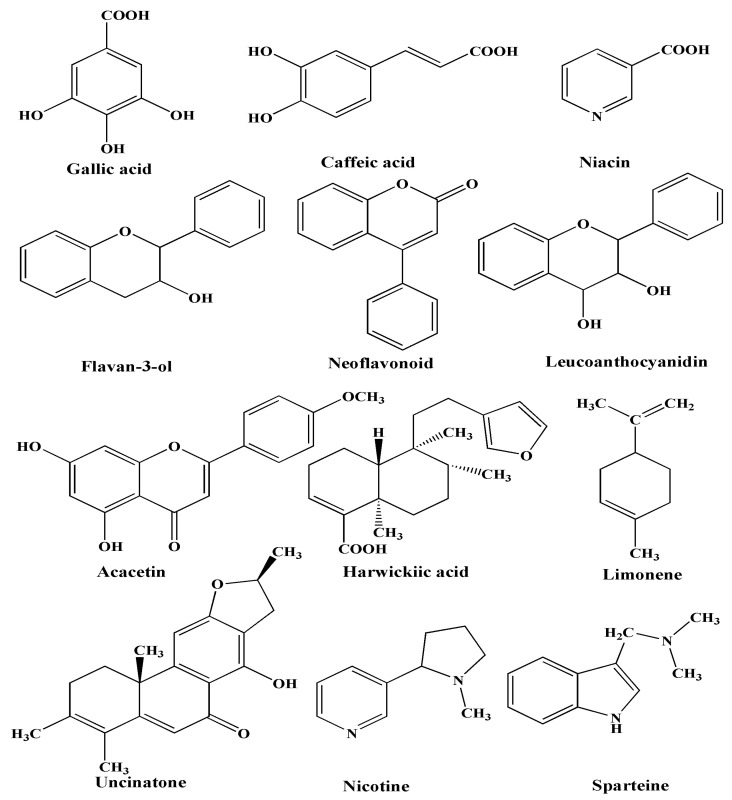
Phytochemicals present in *C. inerme* leaves extract [18,19,20].

**Figure 15:**
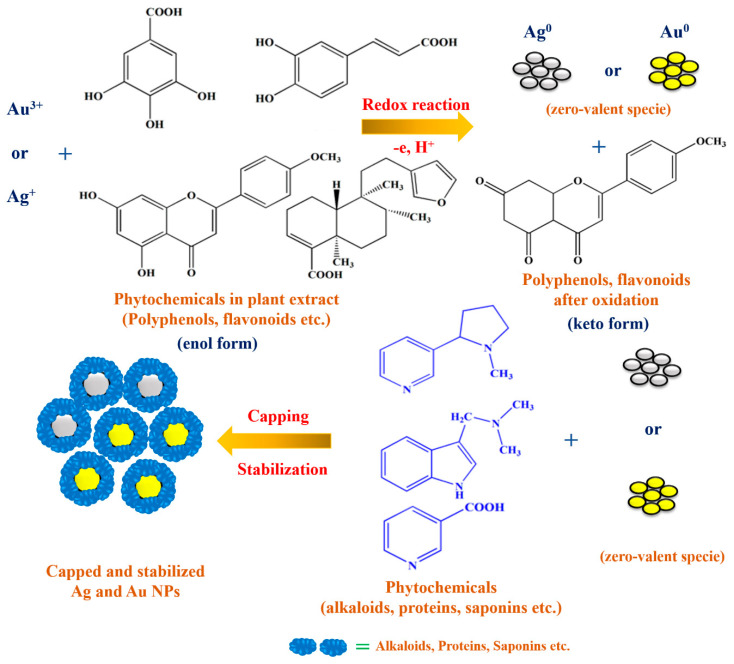
The schematic illustration displays the anticipated mechanism for green synthesis of CI-Au NPs and CI-Ag NPs using leaves extract of *C. inerme*.

It has been reported that *C. inerme* leaves extract possesses numerous biological active phytochemical compounds such as flavonoids, phenolics, alkaloids, terpenoids, anthraquinones, carbohydrates, saponins, and tannins, as shown in Figure 14 [18,19,20]. In the present synthesis processes, metal ions are reduced to metal nanoparticles with only the leaves extract as the other reactant. This suggests that some components of the leaves extract act as reducing agents in the synthetic reaction. At the same time, the CI-Au and CI-Ag NPs show much better antioxidant, antibacterial and antimycotic activities compared with commercial Au and Ag NPs. Together with the FTIR results, we can conclude that some bioactive components from the leaves extract do remain on the surfaces of the CI-Au and CI-Ag NPs. During synthesis, gold and silver salts in leaves extract first dissociated into their ions, such as Au^3+^ and Ag^+^. Phytochemicals, such as flavonoids, phenolics, carbohydrates, cardiac glycosides, and anthraquinones, in the leaves extract can first reduce the metal ions into their zero-valent species. While capping agents, such as terpenoids, tannins, saponins, alkaloids, and proteins, can encapsulate the Au and Ag zero-valent species to stabilize them (Figure 15).

It has been shown that antimicrobial and antioxidant properties can be enhanced by anchoring biocompatible and biologically active molecules to synthesized metal NPs. In the current work, we demonstrate that the benefits of these bioactive components can be simultaneously obtained in a green synthesis process using a plant with bioactive components. In the present case, the adsorbed biologically active phytochemicals are bacteriostatic and fungicidal in nature as they have a substitution of different molecular functionalities (-OH, -NO_2_, -COOH, -SO_3_H, -NH_2_, -CONH_2_, etc.), which play a vital role in various biological activities (Figure 14) [19].

Finally, we have compared the antimicrobial potential of our synthesized CI-Ag, and CI-Au NPs at the concentration of 250 µg/mL (equivalent to 2317.643 µM and 1269.250 µM) with Ag and Au NPs, respectively, synthesized with other plants in Table 1 and Table 2. It can be seen that the green synthesized CI-Au NPs and CI-Ag NPs are highly effective for prohibiting the growth of both Gram-negative bacteria (*E. coli* and *Klebsiella*) than Gram-positive (*S. aureus* and *B. subtilis*) as well as fungi (*A. flavus* and *A. niger*).

**Figure 16:**
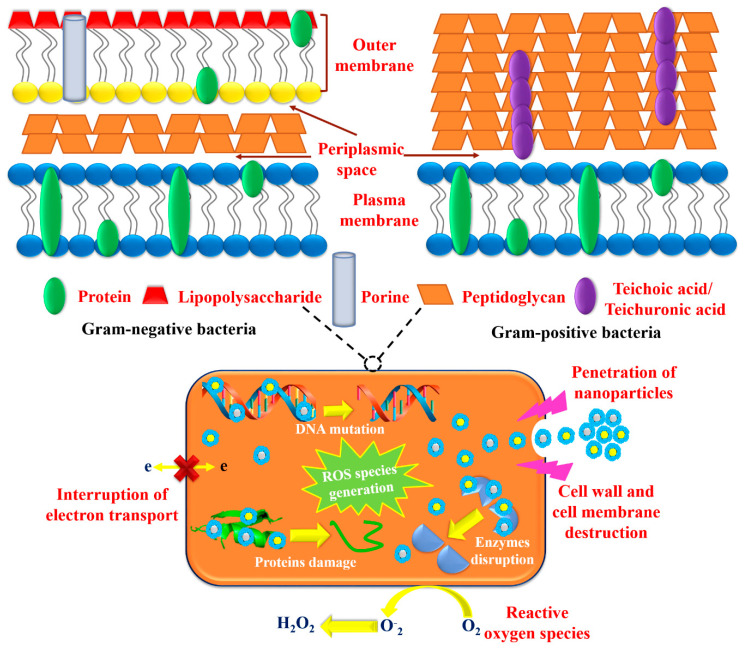
Cell wall comparison of Gram-positive and Gram-negative bacteriological strains.

It has been anticipated from the antibacterial results that green fabricated CI-Au NPs and CI-Ag NPs were found to manifest more excellent growth inhibitory action against *E. coli*, and *Klebsiella* (Gram-negative bacterial strains) than *S. aureus* and *B. subtilis* (Gram-positive bacterial strains) (Figure 6b). This attributes to the fact of differences in chemical composition and structure of their cell wall. The cell wall of Gram-negative bacterial strains has a thin layer of peptidoglycan with an extra outer covering layer of lipopolysaccharide called periplasm. On the other hand, the cell wall of Gram-positive bacterial strains has a thick peptidoglycan layer, as shown in Figure 16 [37]. The literature demonstrates that Gram-negative bacterial strain, i.e., *E. coli*, has ~8 nm thick peptidoglycans layer and 1–3 µm thick lipopolysaccharides layer as well in their cell wall. While Gram-positive bacterial strain, i.e., *S. aureus*, has much thick layer (~80 nm) of peptidoglycans with covalently-attached teichuronic and teichoic acid. Due to the thinner layer of peptidoglycans in the cell walls, Gram-negative bacterial strains are highly vulnerable to the penetration of NPs and their antibacterial action than Gram-positive bacteria. Another factor for Gram-negative bacterial strains to exhibit high sensitivity towards NPs is the existence of lipopolysaccharides coatings outside their cell as these coatings are negatively charged. These lipopolysaccharides coatings have a greater affinity towards NPs with positive surface charge [37]. Hence, due to these above factors, green fabricated CI-Au NPs and CI-Ag NPs are proved to demonstrate significant effectiveness against Gram-negative bacterial strains.

Antimicrobial activities of Au NPs and Ag NPs have been attributed either by their physical or oxidative vandalization or by both to the microbial cells [36,38]. It has been reported that gold and silver NPs possess a higher affinity to proteins and tend to bind to the surface proteins of cells [38]. As per the hard-soft acid−base theory, Au NPs and Ag NPs possess a higher affinity for phosphorus and sulfur moieties of proteins. In addition, Ag and Au also have a tendency to form bonds with nitrogen (i.e., Ag−N and Au−N bonds) and with oxygen (i.e., Ag−O) moieties of proteins [39]. The drastic changes take place in membrane permeability upon the binding of Au NPs and Ag NPs with the cell surface proteins, which leads to depletion in the level of intracellular ATP and the dissipation of proton motive force that results in microbial cells to demise [36,40]. As well, Ag and Au possess higher redox potential [EH^0^ (Ag^+^/Ag^0^) = 0.8 V] and [EH^0^ (Au^+^/Au^0^) = 1.83 V] respectively. Their higher redox potential does oxidative disintegration of lipopolysaccharides and cell surface proteins, which further leads to cell membrane destruction and pore formation on the cell membrane due to which seepage of intracellular contents occurs [36,37,38,39]. Moreover, this pore formation on cell membranes also causes Au and Ag NPs internalization, which vandalizes the intracellular proteins and nucleic acids due to NPs interaction with them [36,41]. Recent reports on antimicrobial mechanism of metal NPs also proposed that Au and Ag NPs demonstrate microbicidal activity due to the production of oxidative stress in microbial cells by them leading to the generation of ROS species, which subsequently cause drastic destruction to cells such as cell membrane impairment, seepage of cellular material, loss of respiratory activity, as well as DNA damage leading to cell demise [36,38]. 

The outcomes of this research work recommend that green synthesized CI-Au NPs and CI-Ag NPs exhibit excellent antimicrobial activity because of the extraordinary colloidal stability of phytochemicals capped NPs. The intracellular ROS investigations have been affirmed that annihilation of microbial’s cell membrane and following cell demise by CI-Au NPs and CI-Ag NPs was caused by the generation of ROS species and membrane permeabilization. Further, FT-IR spectroscopic study disclosed alterations in chemical compositions of cell’s biological molecules (fatty acids, carbohydrates, and proteins) during the assassination process of microbial cells. In view of the uniformity and consistency in results we propose that antimicrobial propensity presented by CI-Au NPs and CI-Ag NPs is the combining effect of physical and oxidative destructions with the following cellular changes: (1) binding of CI-Au NPs and CI-Ag NPs with lipopolysaccharides and cell surface proteins leads to the cell membrane destruction; (2) deterioration of microbial cell membrane increased the membrane permeability, consequently instigated the seepage of intracellular biomolecular functionality; (3) over the cellular antioxidant defense system, generation of intracellular ROS species impaired the microbial cells, which leads to cell demise.

## 5. Conclusions

Current research work demonstrated the successful fabrication of CI-Au and CI-Ag NPs with inherited biomedical functions of *C. inerme* extract via an environment-friendly green approach. Green synthesized NPs were successfully characterized using different characterization techniques such as XRD, UV-Visible, FTIR, TEM, EDX, and DLS. Results demonstrate that green synthesized CI-Au NPs and CI-Ag NPs have much better antioxidant, antibacterial, antimycotic performance comparing to commercial Au and Ag NPs functionalized with dodecanethiol and PVP, respectively. Further, they appeared more biocompatible than commercial NPs. The synthesized NPs exhibited enhanced biological activities due to the synergetic addition of biologically active adsorbed phytochemicals. Hence, this research work further has proven that environment-friendly and modest green synthesis of CI-Au NPs and CI-Ag NPs with enhanced and/or additional biomedical functionalities employing leaf extract of *C. inerme* would be an economical and viable substitute to conventional chemical procedures. 

## Figures and Tables

**Table 1:** Comparison of antimicrobial activities of green synthesized CI-Ag NPs with reported Ag NPs synthesized with other plants.

Materials (NPs)	Size (nm)	Plant Used	Antimicrobial Properties			References
			Species	Conc. of NPs	ZOIs	
CI-Ag	2–10	*C. inerme*	*E. coli*	250 µg/mL	17	This work
Ag	8–20	*D. bulbifera*	*E. coli*	500 µg/mL	15	[42]
Ag	8–50	*Allium ampeloprasum*	*E. coli*	300 µg/mL	13	[43]
Ag	20	Umbrella	*E. coli*	250 µg/mL	16	[44]
Ag	36–74	*Trianthema decandra*	*E. coli*	10 mg/mL	15.5	[45]
CI-Ag	2–10	*C. inerme*	*S. aureus*	250 µg/mL	14	This work
Ag	10–20	Green and black tea	*S. aureus*	1 mg/mL	19–21	[46]
Ag	10–20	*Zingiber officinale*	*S. aureus*	0.1 mg/mL	6.5	[47]
Ag	8–50	*Allium ampeloprasum*	*S. aureus*	300 µg/mL	8	[43]
Ag	20	Umbrella	*S. aureus*	250 µg/mL	12.7	[44]
Ag	36–74	*Trianthema decandra*	*S. aureus*	10 mg/mL	13.5	[45]
CI-Ag	2–10	*C. inerme*	*K. pneumoniae*	250 µg/mL	21	This work
Ag	8–20	*D. bulbifera*	*K. pneumoniae*	500 µg/mL	15	[42]
Ag	20	Umbrella	*K. pneumoniae*	250 µg/mL	13.1	[44]
Ag	50	*Aesculus hippocastanum*	*K. pneumoniae*	100 µg/mL	12.5	[48]
CI-Ag	2–10	*C. inerme*	*B. subtilis*	250 µg/mL	15	This work
Ag	37	*E. scaber*	*B. subtilis*	1 mg/mL	16	[49]
Ag	20–25	*P. guajava*	*B. subtilis*	300 µg/mL	19	[50]
Ag	10–20	*Zingiber officinale*	*B. subtilis*	0.1 mg/mL	0	[47]
Ag	36–74	*Trianthema decandra*	*B. subtilis*	10 mg/mL	12	[45]
CI-Ag	2–10	*C. inerme*	*A. flavus*	250 µg/mL	22	This work
Ag	37	*E. scaber*	*A. flavus*	1 mg/mL	12	[49]
CI-Ag	2–10	*C. inerme*	*A. niger*	250 µg/mL	17	This work
Ag	20–25	*P. guajava*	*A. niger*	300 µg/mL	18.79	[50]

**Table 2:** Comparison of antimicrobial activities of green synthesized CI-Au NPs with reported Au NPs synthesized with other plants.

Materials (NPs)	Size (nm)	Plant Used	Antimicrobial Properties			References
			Conc. of NPs	Species	ZOIs	
CI-Au	3–9	*C. inerme*	*E. coli*	250 µg/mL	16	This work
Au	15.6	*Plumeria alba*	*E. coli*	400 µg/mL	16	[51]
Au	2.7–38.7	*Achillea wilhelmsii*	*E. coli*	300 µg/mL	0	[52]
Au	20–140	*Citrullus lanatus*	*E. coli*	1000 µg/mL	9.23	[53]
Au	33–65	*Trianthema decandra*	*E. coli*	10 mg/mL	9.5	[45]
Au	40–45	*Gundelia tournefortii*	*E. coli*	2 mg/mL	9.8	[54]
Au	40–45	*Falcaria vulgaris*	*E. coli*	4 mg/mL	8.6	[55]
Au	40–45	*Allium saralicum*	*E. coli*	4 mg/mL	10.8	[56]
CI-Au	3–9	*C. inerme*	*S*. *aureus*	250 µg/mL	13	This work
Au	20–140	*Citrullus lanatus*	*S*. *aureus*	1000 µg/mL	0	[53]
Au	33–65	*Trianthema decandra*	*S*. *aureus*	10 mg/mL	14.5	[45]
Au	40–45	*Gundelia tournefortii*	*S*. *aureus*	2 mg/mL	11.2	[54]
Au	40–45	*Falcaria vulgaris*	*S*. *aureus*	4 mg/mL	13	[55]
Au	40–45	*Allium saralicum*	*S*. *aureus*	4 mg/mL	11.6	[56]
CI-Au	3–9	*C. inerme*	*B. subtilis*	250 µg/mL	14	This work
Au	2.7–38.7	*Achillea wilhelmsii*	*B. subtilis*	300 µg/mL	11	[52]
Au	33–65	*Trianthema decandra*	*B. subtilis*	10 mg/mL	9.5	[45]
Au	40–45	*Gundelia tournefortii*	*B. subtilis*	2 mg/mL	14.2	[54]
Au	40–45	*Falcaria vulgaris*	*B. subtilis*	4 mg/mL	14	[55]
Au	40–45	*Allium saralicum*	*B. subtilis*	4 mg/mL	14.2	[56]
CI-Au	3–9	*C. inerme*	*A. niger*	250 µg/mL	15	This work
Au	12–22	*Brassica oleracea*	*A. niger*	50 µg/mL	9	[57]
CI-Au	3–9	*C. inerme*	*A. flavus*	250 µg/mL	20	This work
Au	12–22	*Brassica oleracea*	*A. flavus*	50 µg/mL	9	[57]

## Supplementary Materials

The following are available online at https://www.mdpi.com/2218-273X/10/6/835/s1, Figure S1: The particle size distribution histogram of green synthesized (a) CI-Au and (b) CI-Ag NPs from TEM, Figure S2: Hemolysis activity of different concentrations of green synthesized CI-Au, and CI-Ag NPs in comparison to C. inerme leaves extract, Au NPs, and Ag NPs.
