# Supplementary material for: Green Synthesis of Gold and Silver Nanoparticles Using Leaf Extract of Clerodendrum inerme; Characterization, Antimicrobial, and Antioxidant Activities
Source: Biomolecules. 2020 May 29;10(6):835. doi: 10.3390/biom10060835 (PMC7356939; doi:10.3390/biom10060835)
Supplement: Supplementary file 1 [file biomolecules-10-00835-s001.pdf]

Supplementary Material

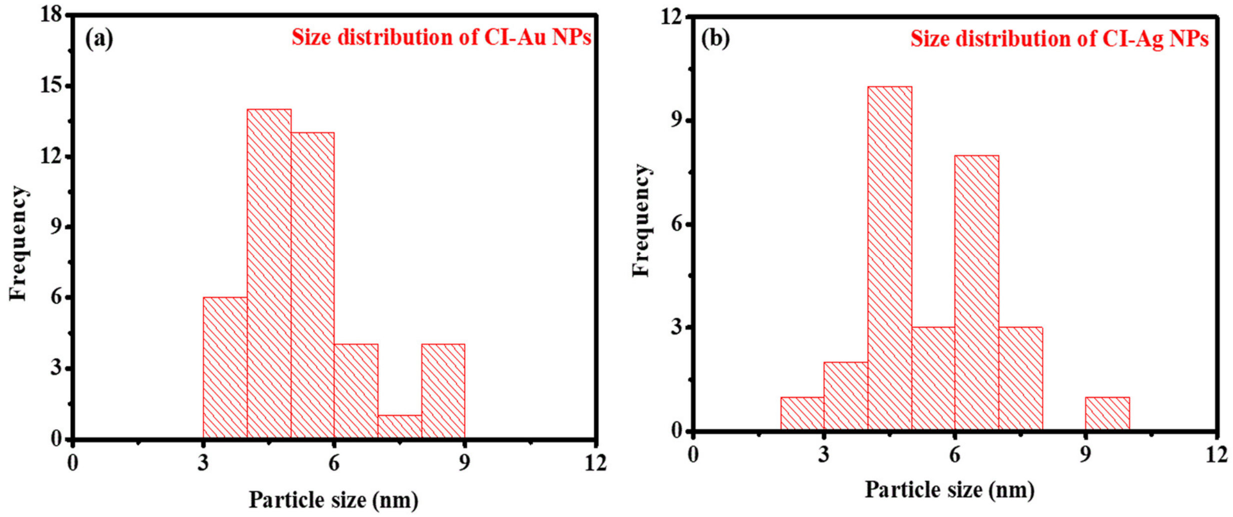

Figure S1. The particle size distribution histogram of green synthesized (a) CI-Au and (b) CI Ag NPs from TEM.

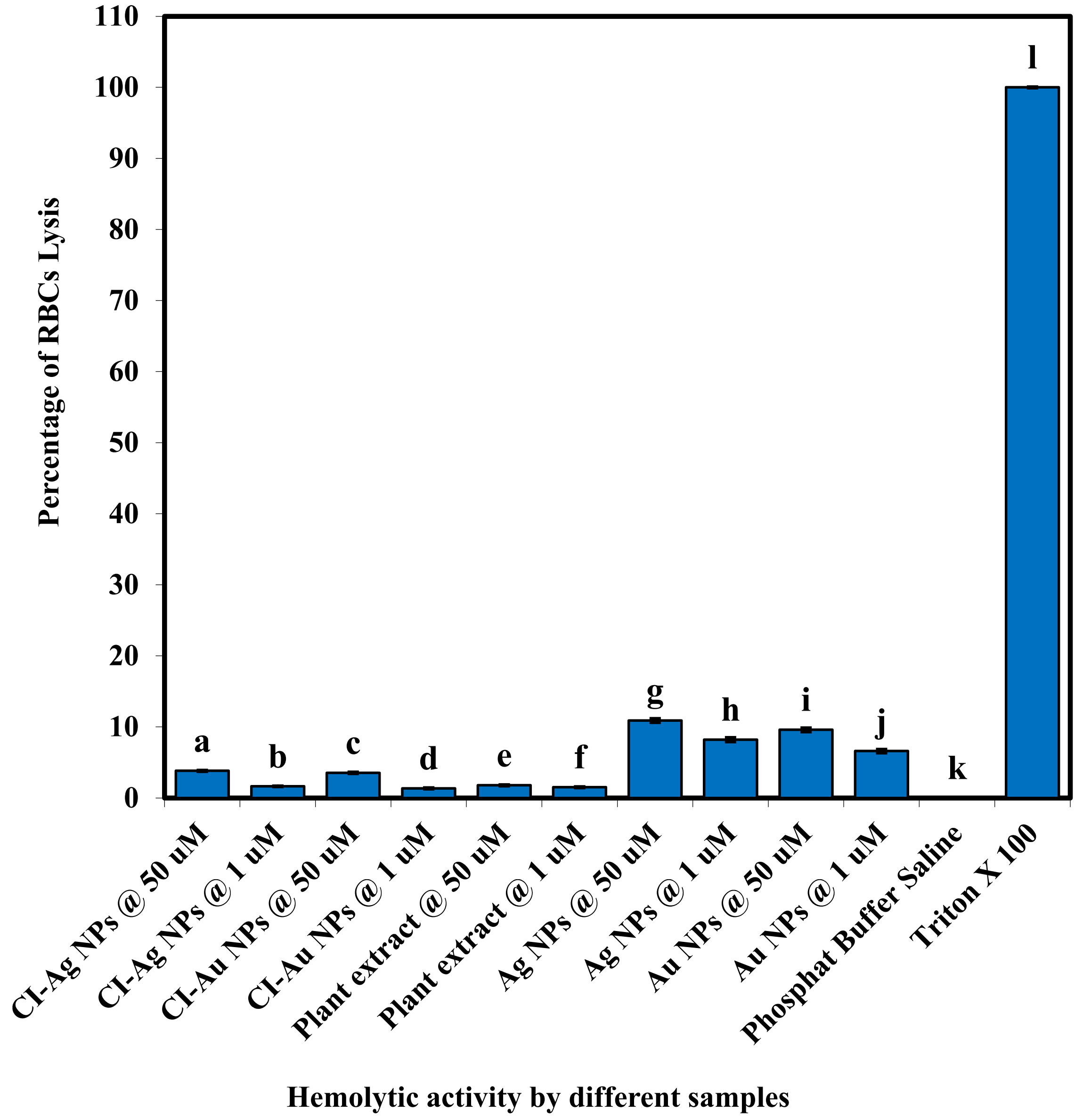

Figure S2. Hemolysis activity of different concentrations of green synthesized CI-Au and CI-Ag NPs in comparison to *C. inerme* leaves extract, Au NPs, and Ag NPs. (Note; Tukey based heterogeneous lower-case letters represent significant statistical pairs)
